# Chronic Administration with FAD012 (3,5-Dimethyl-4-hydroxycinnamic Acid) Maintains Cerebral Blood Flow and Ameliorates Swallowing Dysfunction After Chronic Cerebral Hypoperfusion in Rats

**DOI:** 10.3390/ijms26073277

**Published:** 2025-04-01

**Authors:** Takashi Asano, Hirokazu Matsuzaki, Meiyan Xuan, Bo Yuan, Jun Takayama, Takeshi Sakamoto, Mari Okazaki

**Affiliations:** 1 Laboratory of Pharmacology, Faculty of Pharmaceutical Sciences, Josai University, Saitama 350-0295, Japan; tasano0907@gmail.com (T.A.); ma-tsu@josai.ac.jp (H.M.); yuanbo@josai.ac.jp (B.Y.); 2 Laboratory of Organic and Medicinal Chemistry, Faculty of Pharmaceutical Sciences, Josai University, Saitama 350-0295, Japan; genbien@josai.ac.jp (M.X.); takayama@josai.ac.jp (J.T.); sakamoto@josai.ac.jp (T.S.)

**Keywords:** 3,5-dimethyl-4-hydroxycinnamic acid (ferulic acid derivative 012, FAD012), ferulic acid (FA), cerebral hypoperfusion (2VO), dysphagia (swallowing dysfunction), oxidative stress, substance P (SP), nigrostriatal dopamine-SP system

## Abstract

Dysphagia is a serious complication of stroke, yet effective pharmacological treatments remain limited. This study investigated the effects of FAD012 (3,5-dimethyl-4-hydroxy cinnamic acid), a synthetic derivative of ferulic acid (FA), on cerebral damage and swallowing dysfunction in a rat model of bilateral common carotid artery occlusion (2VO). Sprague–Dawley rats were orally administered FAD012 (3 or 10 mg/kg), FA (10 mg/kg), or 0.5% carboxymethyl cellulose (CMC, suspension vehicle) starting one week before 2VO. Two weeks after 2VO surgery, which was performed under isoflurane anesthesia, reflex swallowing was assessed by electromyographic recordings of the mylohyoid muscle under urethane anesthesia. Two weeks after 2VO, cerebral blood flow (CBF) declined to approximately 40% of baseline, and the number of reflex swallowing responses was significantly reduced in the CMC group. Additionally, 2VO induced O_2_^−^ production, apoptotic cell death in the striatum, and a reduction in tyrosine hydroxylase expression. Substance P (SP) levels in the laryngopharyngeal mucosa, positively regulated by dopaminergic signaling in the basal ganglia, also decreased. FAD012 (10 mg/kg) effectively prevented the 2VO-induced reduction in CBF, enhanced the reflex swallowing, and preserved the dopamine-SP system. Notably, FAD012 exerted significantly stronger effects than FA at the same dose. These findings suggest that FAD012 maintains CBF under cerebral hypoperfusion and enhances the swallowing reflex by maintaining neuronal function in the striatal and laryngopharyngeal regions of 2VO rats.

## 1. Introduction

Stroke is a leading cause of mortality worldwide, with cerebral infarction accounting for approximately 60% of stroke-related deaths [1,2]. Beyond its high mortality rate, stroke often results in long-term disabilities, including cognitive impairments, motor deficits, and sensory disturbances [3]. Among these, dysphagia (difficulty in swallowing) is particularly severe, significantly reducing quality of life and increasing the risk of malnutrition, aspiration pneumonia, and choking [4,5]. Post-stroke dysphagia affects 40% to 60% of survivors, with up to 50% continuing to experience symptoms for six months after the stroke, particularly in regions where higher estimates have been reported [6]. These challenges underscore the need for effective therapeutic and preventive strategies [7].

The current gold standard for acute ischemic stroke treatment is thrombolytic therapy using tissue plasminogen activator (t-PA), which restores cerebral blood flow by dissolving clots [8,9]. However, only 5% to 8% of patients are eligible for t-PA due to its narrow 4.5 h therapeutic window and strict inclusion criteria. Moreover, even among eligible patients, t-PA carries a substantial risk of hemorrhagic complications, with a modest success rate of about 30% [2]. These limitations underscore the urgent need for preventive pharmacological interventions to mitigate stroke severity and its complications, particularly in high-risk populations.

Ferulic acid (FA), a naturally occurring phenolic compound found in rice bran and coffee beans, has garnered attention for its neuroprotective properties. With a low toxicity profile and diverse pharmacological effects, including antioxidant, anti-apoptotic, and vasodilatory activities [10], FA has shown potential in mitigating stroke-related complications [11,12]. Our previous study demonstrated that repeated administration of FA reduces oxidative stress and protects dopaminergic neurotransmission in the striatum, preserving the nigrostriatal dopamine-SP system and improving swallowing function in a rat model of cerebral ischemia induced by bilateral common carotid artery occlusion (2VO) [13], a well-established model of post-stroke dysphasia [14]. Reflex swallowing is controlled by the swallowing central pattern generator (CPG) in the medulla oblongata, which is activated by sensory input from afferent fibers, such as the superior laryngeal and glossopharyngeal nerves, in response to laryngopharyngeal mechanical and chemical stimulation [15]. Dopamine in the nigrostriatal system plays a critical role in maintaining the swallowing reflex by increasing substance P (SP) levels in laryngopharyngeal sensory fibers, which are essential for initiating the swallowing reflex [16,17,18,19]. Post-stroke basal ganglia infarcts often impair the swallowing reflex and cough reflex, leading to silent aspiration, due to reduced sensory input to the CPG [20]. Basal ganglia damage diminishes dopaminergic transmission and SP levels in the striatum, contributing to oropharyngeal dysphagia [21]. Similar mechanisms are implicated in Parkinson’s disease and aging-related dysphagia [21,22]. We previously demonstrated that FA reduced oxidative damage and maintained tyrosine hydroxylase (TH) expression in striatum, thereby protecting the nigrostriatal dopamine-SP system in the 2VO rats [13]. These effects suggest the therapeutic potential of FA in preventing and treating stroke-induced dysphagia.

**Figure 1:**
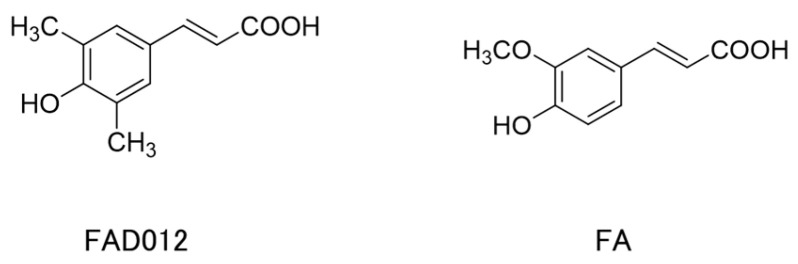
Chemical structures of 3,5-dimethyl-4-hydroxycinnamic acid (ferulic acid derivative 012; FAD012) and ferulic acid (FA).

Building on the cerebroprotective potential of FA, we developed 3,5-dimethyl-4-hydroxycinnamic acid (ferulic acid derivative 012; FAD012), a synthetic FA derivative, to enhance its pharmacological profile as a prophylactic drug intended for chronic administration to mitigate the impact of unexpected ischemic stroke events. Our recent study confirmed the good tolerability and low toxicity of long-term FAD012 administration [23]. FAD012 was designed with improved lipophilicity for blood–brain barrier (BBB) permeability, which makes it a promising candidate for prophylactic treatment in cerebral ischemia. Structurally, FAD012 differs from FA by replacing the methoxy group with a methyl group on the aromatic ring, aimed to enhance both lipophilicity and the electron-donating ability of the phenolic hydroxyl group (Figure 1). Our research to date has shown that in comparison to FA, FAD012 exhibits superior potency across a range of beneficial antioxidant and cytoprotective activities, while maintaining a low toxicity profile similar to that of FA [23,24]. Cellular assays using rat brain microvascular endothelial cells (RBMVECs) revealed that FAD012 provides markedly superior protection against hydrogen peroxide-induced oxidative damage compared to FA. Consistently, the in vivo study showed that FAD012 maintains cerebral blood flow (CBF) and mitigates ischemic brain injury in a rat middle cerebral artery occlusion following reperfusion (MCAO/Re) models, suggesting its ability to preserve endothelial integrity under ischemic conditions [23].

This study aimed to further evaluate FAD012 as a more effective prophylactic agent against oropharyngeal dysphagia in a rat model of 2VO, providing insights into the development of preventive and therapeutic approaches for both acute and long-term complications of stroke. To assess the neuroprotective effects of FAD012, neuronal oxidative stress, apoptosis, and TH expression in the striatum of 2VO rats were histochemically evaluated. Furthermore, expression levels of SP in both the striatum and laryngopharyngeal region were analyzed.

## 2. Results

### 2.1. Body Weights and Survival Rates

FAD012 (3 or 10 mg/kg), FA (10 mg/kg), and 0.5% carboxymethyl cellulose in saline solution (CMC, suspension vehicle) were orally administered daily starting 1 week prior to 2VO. The 2VO resulted in significant body weight loss and reduced survival rates in rats (Table 1). Neither FAD nor FA influenced 2VO-induced body weight loss. Survival rates tended to be higher in rats treated with FAD012 and FA, though the difference was not statistically significant.

### 2.2. Changes in Cerebral Blood Flow (CBF) After Bilateral Common Carotid Artery Occlusion (2VO)

**Figure 2:**
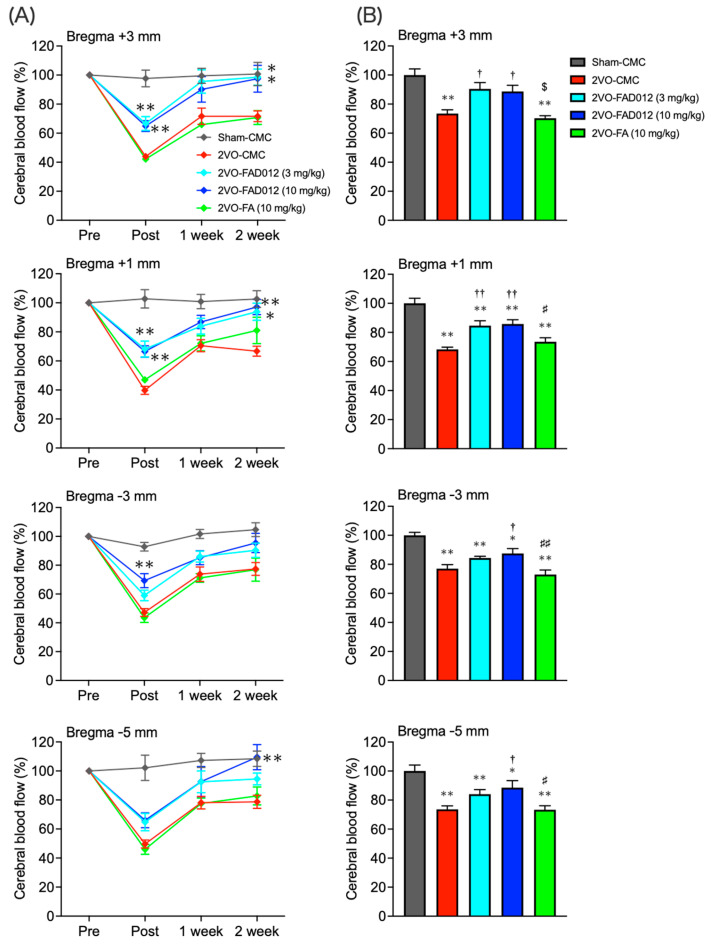

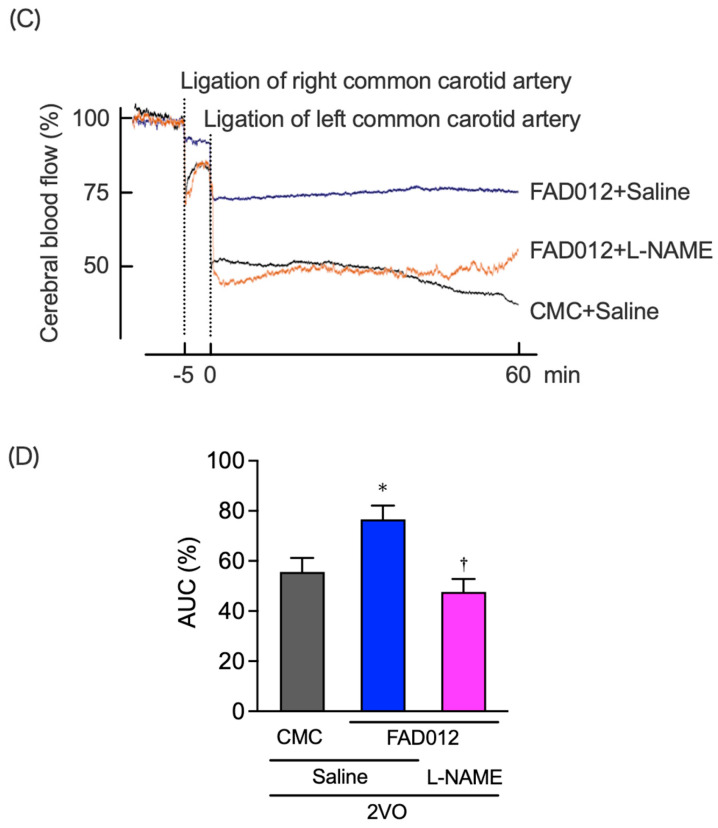
Part 1. See next page for continuation. Effects of chronic treatment with FAD012 on CBF in rats subjected to 2VO. (**A**) Temporal changes in surficial CBF at four rostrocaudal cortical levels (+3.0, +1.0, −3.0, −5.0 mm from the bregma and 3.0 mm lateral to the midline) measured using laser Doppler flowmetry 30 min before (Pre) and 30 min after (Post), as well as 1 and 2 weeks after 2VO. Data represent the average values from both hemispheres. The CBF values after 2VO (Post, 1-week, and 2-week) were expressed as a percentage of the average value for Pre. (**B**) Area under the curve (AUC) for CBF was calculated for each group based on the sum of CBF values measured at 2VO post, 1 week, and 2 weeks, with the average value for Sham-CMC group defined as 100%. Data are expressed as means ± S.E.M., *n* = 6–12 per group. *, ** *p* < 0.05, 0.01 vs. Sham-CMC group; ^†^, ^††^ *p* < 0.05, 0.01 vs. 2VO-CMC group; ^#^, ^##^ *p* < 0.05, 0.01 vs. 2VO-FAD012 group; ^$^ *p* < 0.05; vs. 2VO-FAD (3 mg/kg). Figure 2. Part 2. Continued from the previous page. (**C**) Representative CBF recording measured before and after 2VO in rats that received CMC or FAD012 (10 mg/kg) for 2 weeks. N^G^-nitro-L-arginine methyl ester (L-NAME) was administered intraperitoneally 30 min before 2VO. The CBF value after 2VO was expressed as a percentage of the average value for 5 min before 2VO. (**D**) AUC for CBF was calculated for each group using the percentage values of CBF over the 60-min period following 2VO. Saline was administered intraperitoneally as a control for the L-NAME treatment. Data are expressed as means ± S.E.M., *n* = 6 per group. * *p* < 0.05 vs. Saline-CMC group; ^†^ *p* < 0.05, vs. Saline-FAD012 group.

Recently, we demonstrated that FAD012 preserved vascular endothelial viability and maintained endothelial nitric oxide synthase (eNOS) expression, contributing to the restoration of CBF during MCAO in rats [23]. To further investigate its effects on cerebral circulation under hypoperfusion, frontoparietal cortical CBF was continuously monitored at four rostrocaudal levels across both hemispheres for 2 weeks post-2VO using a laser Doppler blood flow meter. As shown in Figure 2A, CBF in the 2VO-CMC group decreased to around 50% immediately post-2VO and gradually recovered to 70% over 14 days. Similar changes were observed in the FA group. However, in the FAD012-treated group, the reduction in CBF was continuously mitigated throughout the 2-week period. The total CBF value from the pre-ischemic phase to 2 weeks post-2VO decreased to approximately 70% in the 2VO-CMC group, while the decrease was significantly attenuated in the FAD012-treated group (Figure 2B).

The role of NO in the action of FAD012 to maintain CBF during ischemia was evaluated using the specific NOS inhibitor N^G^-nitro-L-arginine methyl ester (L-NAME), which is commonly used to inhibit NO production and evaluate the role of NO signaling. Intraperitoneal administration of L-NAME (30 mg/kg) 30 min prior to 2VO completely abolished the CBF-maintaining effect of FAD012 (Figure 2C,D).

### 2.3. 2VO-Induced Swallowing Dysfunction

**Figure 3:**
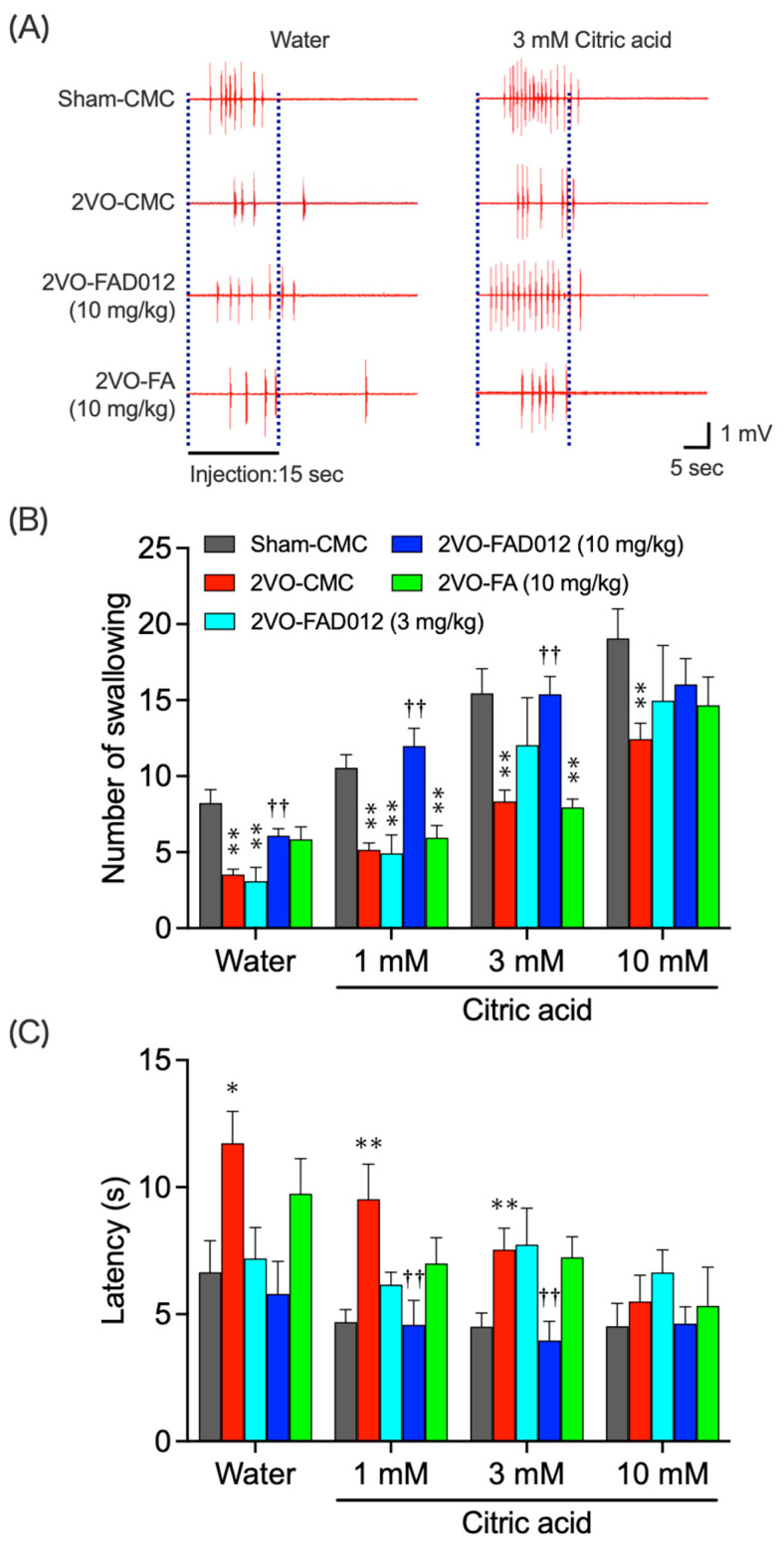
Effects of chronic treatment with FAD012 on swallowing reflex in 2VO rats. (**A**) Representative mylohyoid EMG recordings showing swallowing reflex responses elicited by distilled water or citric acid (1–10 mM) in each group at 14 days post-2VO. (**B**) Quantification of the mean number of swallowing events. (**C**) Latency for the first swallowing response in each group. The data are represented as means ± S.E.M. (*n* = 6–12 per group). *, ** *p* < 0.05, 0.01 vs. Sham-CMC group, ^††^ *p* < 0.01 vs. 2VO-CMC group.

To assess the effect of FAD012 on reflex swallowing in 2VO rats, EMG activity of the mylohyoid muscle was recorded during swallowing induced by the infusion of water or citric acid near the epiglottis. Figure 3A shows typical EMG recordings from each group. In the sham-CMC group, water stimulation elicited multiple swallowing reflexes, with citric acid inducing more frequent and shorter-latency responses. The 2VO-CMC group showed significantly diminished responses to both stimuli. In comparison, rats treated with 10 mg/kg FAD012 demonstrated near-normal swallowing reflexes, whereas FA at the same dose had no significant effect. Quantified data in Figure 3B,C showed that 2VO significantly reduced the number of swallowing events and prolonged swallowing latency. FAD012 treatment restored the number of swallowing events and normalized latency, whereas similar effects were not observed in the FA-treated group.

### 2.4. Oxidative Stress and Apoptotic Cell Death in the Striatum

**Figure 4:**
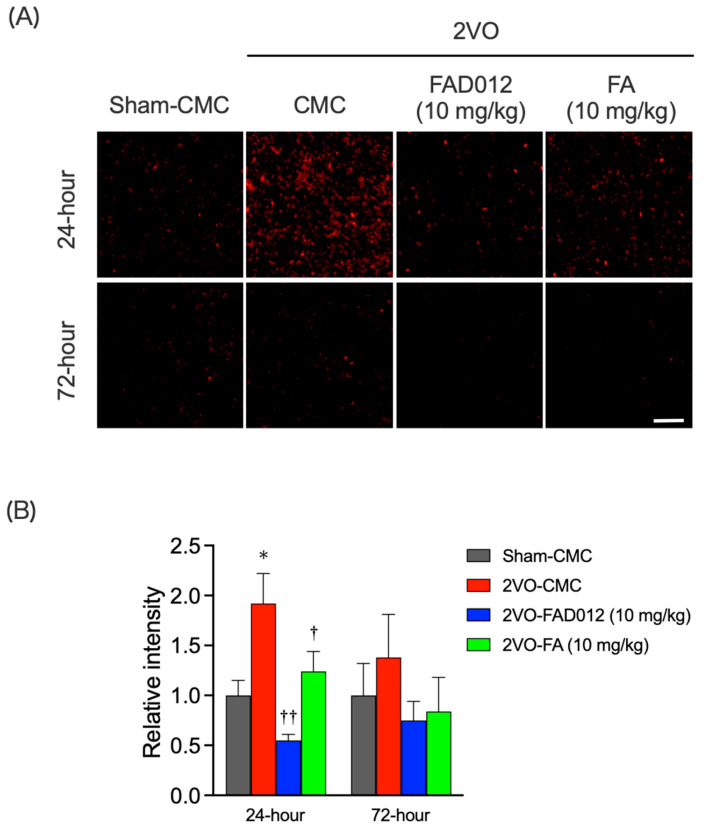
Effects of chronic treatment with FAD012 on striatal oxidative stress after 2VO. Representative results of dihydroethidium (DHE) staining for superoxide production at 24 and 72 h post-2VO in the striatum from rats in each group (**A**), scale bar = 200 µm; Fluorescence intensity of oxidized DHE was quantified using imaging software focused in the relevant areas (**B**). The values of fluorescence intensity of each group are represented as means ± S.E.M. relative to those of 2VO-CMC group; *n* = 4–9. * *p* < 0.05 vs. Sham-CMC group, ^†^, ^††^ *p* < 0.05, 0.01 vs. 2VO-CMC group.

Next, we assessed oxidative stress and cell death in the striatum 24 or 72 h post-2VO. DHE staining was used to quantify O_2_^−^ production, a marker of oxidative stress. At 24 h after the 2VO, O_2_^−^ production in the striatum was significantly increased in the 2VO-CMC group, approximately doubling compared to the Sham-CMC group, whereas no such increase was observed in the FAD012-treated group (Figure 4A). Quantitative analysis confirmed that O_2_^−^ generation was significantly suppressed in the FAD012 group (0.55 ± 0.06) compared to the 2VO-CMC group (*p* < 0.01) (Figure 4B). FA at the same dose also exhibited significant antioxidant effects (1.24 ± 0.20) (*p* < 0.01), although its efficacy was lower than that of FAD012. By 72 h after the 2VO, O_2_^−^ production induced by 2VO had diminished.

**Figure 5:**
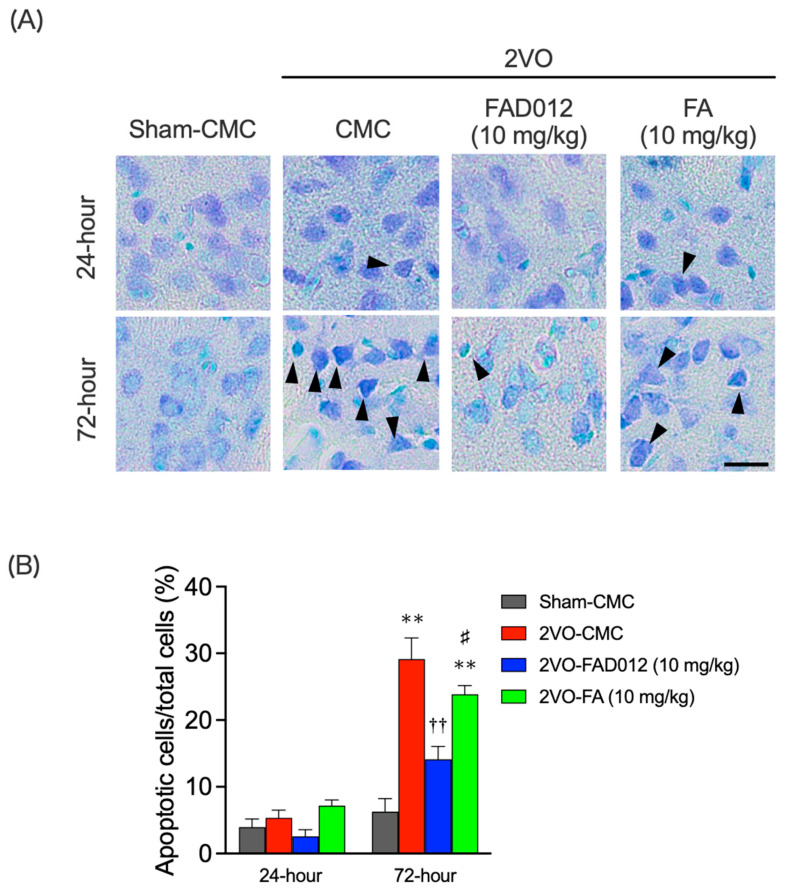
Effects of chronic treatment with FAD012 on apoptotic cell death in the striatum after 2VO. (**A**) Representative microphotographs of Nissl-stained coronal sections from each group at 72 h post-2VO. Black triangles indicate apoptotic cells showing nuclear condensation. Neuronal loss and nuclear condensation are prominently observed in the 2VO-CMC group. Scale bar = 20 µm. (**B**) Quantification of the percentage of darkly stained, condensed neurons in the striatal region. Data are presented as means ± S.E.M. (*n* = 4–9 per group). ** *p* < 0.01 vs. sham-CMC group; ^††^ *p* < 0.01 vs. 2VO-CMC group; ^#^ *p* < 0.05 vs. 2VO-FAD012 group.

Nissl staining 24 h post-2VO revealed no significant cell death in any group. However, by 72 h post-2VO, the numbers of dead cells markedly increased in the 2VO-CMC group (29.15 ± 3.14) (*p* < 0.01), indicating delayed neuronal death due to hypoperfusion. FAD012 treatment significantly reduced nuclear aggregation and suppressed cell death (14.14 ± 1.91) (Figure 5A,B), while FA had no protective effect.

**Figure 6:**
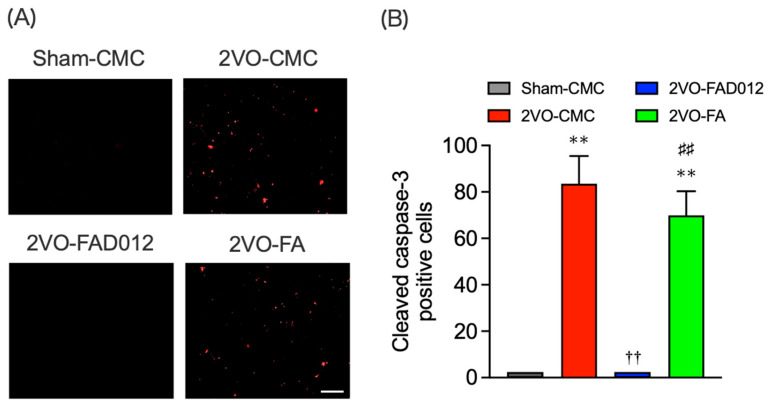
Effects of chronic treatment with FAD012 on apoptotic cell death in the striatum after 2VO. Representative microphotographs of cleaved caspase-3 immunostaining at 14 days post-2VO in the striatum from rats in each group (**A**), scale bar = 100 µm; red fluorescence indicates cleaved caspase-3-positive cells detected by Cy3-conjugated secondary antibody. Quantification of the number of cleaved caspase-3 positive cells was achieved by cell counting in the relevant areas of the rat brains in each group (**B**). The data are represented as means ± S.E.M. from 3–5 rats in each group. ** *p* < 0.01 vs. sham-CMC group. ^††^ *p* < 0.01 vs. 2VO-CMC group. ^##^ *p* < 0.01 vs. 2VO-FAD012 group.

To further investigate striatal apoptosis, immunostaining for cleaved caspase-3 was performed (Figure 6A,B). In the 2VO-CMC group, a significantly high number of cleaved caspase-3-positive cells were detected (83.50 ± 11.95) (*p* < 0.01), indicating extensive apoptosis-like neuronal cell death in the striatum, which correlated with impaired swallowing reflexes two weeks post-2VO. FAD012 treatment completely inhibited caspase-3 activation (*p* < 0.01), effectively preventing apoptosis in the striatum. In contrast, FA failed to exert a significant neuroprotective effect, as the number of cleaved caspase-3-positive cells remained comparable to the 2VO-CMC group.

### 2.5. Tyrosine Hydroxylase (TH) Expression in the Striatum

**Figure 7:**
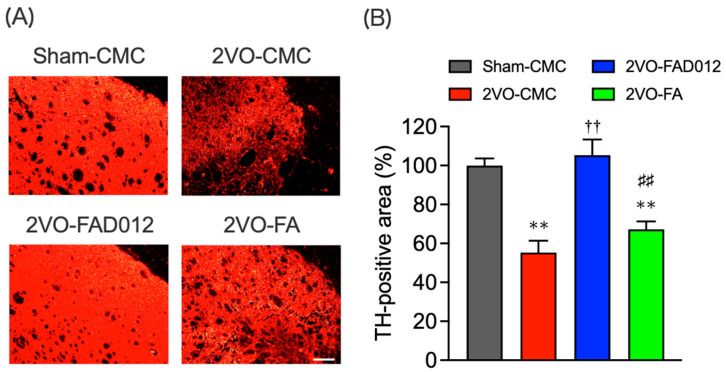
Effects of chronic treatment with FAD012 on expression of tyrosine hydroxylase (TH) in the striatum after 2VO. (**A**) Representative microphotographs of TH immunostaining at 14 days post-2VO in the striatum from rats in each group. Scale bar = 500 µm; (**B**) Quantification of the immunofluorescence was achieved in the relevant areas for rats from each group. The data are represented as means ± S.E.M. from 3–5 rats in each group. ** *p* < 0.01 vs. Sham-CMC group. ^††^ *p* < 0.01 vs. 2VO-CMC group. ^##^ *p* < 0.01 vs. 2VO-FAD012 group.

Dopaminergic neurotransmission in the striatum is essential for maintaining the swallowing reflex, primarily by enhancing SP levels in the sensory fibers of the laryngopharynx [25]. Since the 2VO model closely mimics swallowing reflex impairment in a manner similar to that observed in post-stroke patients with dysfunction of the nigrostriatal dopamine-SP system [26,27], we investigated whether oxidative stress and cell death in the striatum contributed to the swallowing function improvement observed in FAD012 treatment. To evaluate dopaminergic neurotransmission damage in the striatum following 2VO, immunostaining for TH, a key enzyme in dopamine synthesis, was performed to assess dopaminergic neuronal damage in the striatum 14 days after 2VO (Figure 7A,B). In the sham-CMC group, TH immunoreactivity was consistently observed throughout the striatum. However, TH expression was reduced in the 2VO-CMC group (55.32 ± 6.05) (*p* < 0.05 vs. Sham-CMC). The chronic administration of 10 mg/kg FAD012 significantly mitigated this reduction in TH expression (105.33 ± 8.04) (*p* < 0.01 vs. 2VO-CMC), demonstrating a substantially greater effect than FA, which showed little to no effect (Figure 7B).

### 2.6. Substance P (SP) Expression in the Striatum and Laryngopharyngeal Region

**Figure 8:**
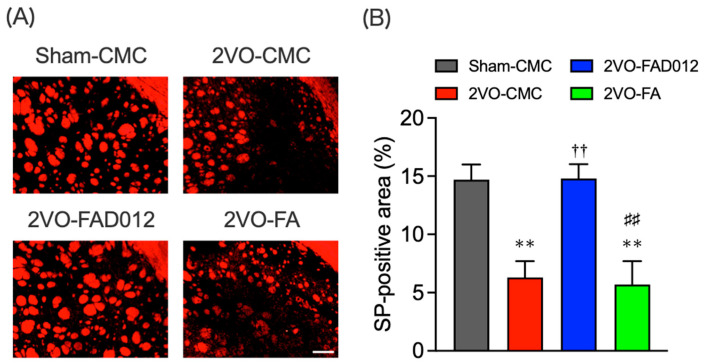
Effects of chronic pretreatment with FAD012 on expression of substance P (SP) in the striatum after 2VO. Representative microphotographs of SP immunostaining at 14 days post-2VO in the striatum of rats from each group (**A**), scale bar = 500 µm; Quantification of the immunofluorescence was achieved in the relevant brain areas from rats in each group (**B**). The data are represented as means ± S.E.M. from 3–5 rats in each group. ** *p* < 0.01 vs. Sham-CMC group. ^††^ *p* < 0.01 vs. 2VO-CMC group. ^##^ *p* < 0.01 vs. 2VO-FAD012 group.

**Figure 9:**
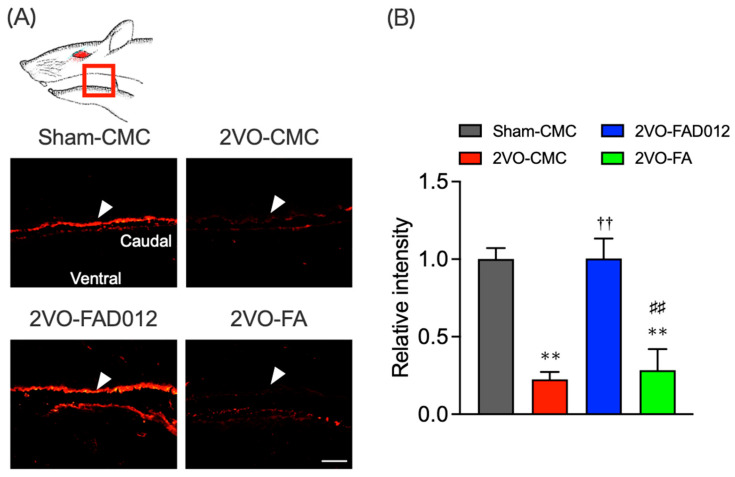
Effects of chronic pretreatment with FAD012 on expression of SP in the laryngopharyngeal region after 2VO. Representative microphotographs of SP immunostaining at 14 days post-2VO in the laryngopharyngeal region (corresponding to the area surrounded by the red frame in the upper illustration) from rats in each group (**A**); white triangles indicate the dorsal mucous membranes, scale bar = 100 µm; Quantification of immunofluorescence was achieved for the relevant regions from rats in each group (**B**). The data are represented as means ± S.E.M. from 3–5 rats in each group. ** *p* < 0.01 vs. Sham-CMC group. ^††^ *p* < 0.01 vs. 2VO-CMC group. ^##^ *p* < 0.01 vs. 2VO-FAD012 group.

Previous studies demonstrated that nigrostriatal dopaminergic transmission positively regulates the expression of SP in the striatum [28] and laryngopharyngeal mucosa [17]. SP expression was evaluated by immunostaining in both striatum (Figure 8) and laryngopharyngeal region 14 days post-2VO (Figure 9). In the 2VO-CMC group, SP expression was significantly reduced compared to the sham group (6.30 ± 1.40) (*p* < 0.05 vs. Sham-CMC). Treatment with 10 mg/kg FAD012 preserved SP expression (14.80 ± 1.23) (*p* < 0.01 vs. 2VO-CMC), whereas no significant difference was observed between the 2VO-CMC rats and the 2VO-FA rats.

In the laryngopharyngeal region, SP expression was also diminished in the 2VO-CMC group (0.23 ± 0.05) (*p* < 0.01 vs. Sham-CMC), particularly in the dorsal mucosa (Figure 9). Treatment with FAD012 maintained SP levels in this region (1.01 ± 0.13) (*p* < 0.01 vs. 2VO-CMC), while FA had no protective effect.

## 3. Discussion

In this study, we investigated the effects of FAD012, a synthetic derivative of FA, on swallowing dysfunction using a rat model of chronic cerebral hypoperfusion induced by 2VO, which mimics dysphagia in the chronic phase of cerebral infarction (Figure 3). Repeated oral administration of FAD012 preserved CBF throughout the 2-week experimental period in 2VO rats (Figure 2A,B). The CBF-maintaining effect of FAD012 was abolished by L-NAME, an NOS inhibitor, suggesting that FAD012 induced vasodilation during ischemia through an NO-mediated mechanism (Figure 2C,D). FAD012 significantly reduced reactive oxygen species (ROS) production (Figure 4) and suppressed delayed neuronal cell death accompanied by caspase-3 activation in the striatum (Figure 5 and Figure 6), suggesting that it inhibited 2VO-induced apoptosis by alleviating ischemic oxidative stress. Notably, TH expression in the striatum was associated with SP levels, a key molecule for triggering the swallowing reflex, in both the striatum and laryngopharyngeal region (Figure 7, Figure 8 and Figure 9). These findings suggest that FA improved swallowing dysfunction by protecting the extrapyramidal dopamine-SP system from ischemia-induced oxidative damage in rats.

Despite the severity of post-stroke dysphagia, effective preventive and therapeutic drugs remain unavailable, underscoring the urgent need for novel treatment strategies [7]. FA, a natural phenolic compound with low toxicity, has shown promising potential as a prophylactic agent against cerebral infarction. Its well-documented antioxidative, anti-inflammatory, anti-apoptotic, and vasoprotective properties contribute to its therapeutic potential. The neuroprotective effects of FA have been well-documented in the context of ischemic brain injury [29] and neurodegenerative diseases, such as Alzheimer’s disease [30,31]. However, its therapeutic efficacy remains limited, indicating its insufficiency as a viable drug candidate. Recent studies, however, highlighted the potential of FA derivatives as enhanced therapeutic agents [32,33]. Novel compounds, such as hmy-paa [32] and NCX2057 [33], demonstrated protective effects against myocardial I/R injury and neuroinflammation by inhibiting ROS production and modulating key inflammatory pathways. These findings suggest that molecular modifications of FA can significantly improve its therapeutic efficacy. In this regard, our recent study demonstrated that FAD012 exhibits similar or slightly stronger radical scavenging and lipid peroxide production inhibition in vitro compared to FA and Trolox as a water-soluble vitamin E analog used as an antioxidant reference [23]. The incorporation of methyl groups at the 3 and 5 positions of the aromatic ring (Figure 1) is believed to enhance the lipophilicity of FAD012, thereby increasing its affinity for lipid-rich cellular membranes. This structural modification is also expected to influence the electronic properties of the molecule, improving the electron-donating capacity and reactivity of the phenolic hydroxyl group. As a result, these changes enhance ROS scavenging activity in biologically relevant microenvironments, such as lipid bilayers.

Mechanical or chemical stimulation of the laryngopharyngeal region triggers sensory input that reflexively activates the medullary CPG, initiating swallowing. In 2VO-CMC rats, stimulation with a high concentration (10 mM) of citric acid induces a swallowing reflex comparable to that in Sham-CMC rats, particularly in terms of swallowing latency, suggesting that the CPG function remains intact following 2VO treatment. This implies that the impairment of the swallowing reflex is not due to CPG dysfunction but rather to weakened sensory input from the laryngopharyngeal region. The preservation of CPG function can be attributed to the intact vertebral arteries, which continue to supply blood to the brainstem even after bilateral common carotid artery occlusion [13]. These findings suggest that swallowing reflex impairment observed in 2VO rats is primarily due to basal ganglia dysfunction rather than CPG failure. Previous studies demonstrated that ACE inhibitors such as perindopril [34] and phosphodiesterase III inhibitors like cilostazol [27] alleviate dysphagia in 2VO-treated rats by protecting the dopamine-SP neural mechanism. These findings, along with the results of the present study, support the validity of the 2VO model in replicating dysphagia observed in the chronic phase of cerebral infarction due to basal ganglia damage.

In this study, prophylactic oral administration of FAD012 (10 mg/kg), initiated one week prior to the 2VO, effectively prevented the 2VO-induced reduction in CBF across the frontoparietal cortex (Figure 2). The neuroprotective effect observed in the striatum (Figure 4, Figure 5, Figure 6, Figure 7 and Figure 8) along with the preservation of CBF under 2VO suggest that CBF-preserving action by FAD012 was not limited to the cortex but also extended to the subcortical structures in the frontoparietal region, potentially contributing to neuroprotection. The ability of FAD012 to sustain CBF was abolished by L-NAME, a NOS inhibitor, suggesting that FAD012 enhances vasodilation under ischemic conditions through an NO-dependent pathway. Koh (2012) reported that FA maintains eNOS expression in cortical tissues of a focal cerebral ischemia model, suggesting that its neuroprotective effects are mediated through sustained eNOS levels, which contribute to improved vascular function and blood flow regulation [35]. However, in the current study, FA at 10 mg/kg did not show a significant effect. Furthermore, our previous report demonstrated that even at a higher dose of 30 mg/kg, FA failed to increase CBF immediately after 2VO, and its ability to preserve CBF remained limited [13]. These results suggest that, unlike FAD012, the cerebroprotective effects of FA are primarily mediated through its antioxidant and anti-inflammatory properties rather than through direct regulation of CBF. This is further supported by the observation that FA, despite its relatively weaker effect, significantly suppressed O_2_^−^ production in the striatum (Figure 4). Recently, we demonstrated that FAD012 protected against hydrogen peroxide-induced oxidative cell death and maintained eNOS expression in RBMVECs, exhibiting significantly greater efficacy than FA [23]. FAD012 also mitigated MCAO/Re-induced damage by preserving CBF through endothelial cell protection in rats [23]. While the precise mechanism by which FAD012 protects endothelial cells remains unclear, these findings suggest that the protective effect on the swallowing reflex is based not only on its antioxidant properties but also on its ability to maintain CBF. This mechanism is considered to play a crucial role in preventing neurodegenerative changes provided by FAD012 in the dopamine-SP system, especially under conditions of sustained cerebral hypoperfusion without reperfusion.

Previous studies demonstrated that cerebral hypoperfusion by 2VO results in global oxidative damage through overproduction of ROS, presumably due to hypoperfusion-induced mitochondrial dysfunction, and triggers the apoptotic process in neuronal cells [36]. FAD012 demonstrated a significantly stronger ability to suppress O_2_^−^ production (Figure 4) and inhibit neuronal apoptosis in the striatum, compared to FA (Figure 5 and Figure 6). Ischemic injury induces neuronal apoptosis primarily through the intrinsic mitochondrial pathway, where oxidative stress disrupts mitochondrial function, leading to cytochrome c release and subsequent activation of caspase-9 and caspase-3 [37]. Caspase-3 is considered to be the critical executioner of nuclear degradation in ischemic neurons and lead to increased mitochondrial membrane permeabilization, DNA fragmentation, and chromatin condensation. FAD012 may inhibit caspase-3 activation by reducing oxidative stress and stabilizing mitochondrial membrane potential, thereby preserving mitochondrial integrity, preventing cytochrome c release, and suppressing mitochondrial permeability transition pore opening, which in turn attenuates apoptotic signaling. FA was shown to directly scavenge ROS and activate multiple antioxidant responses including the induction of antioxidative enzymes such as heme oxygenase (HO-1), superoxide dismutase and catalase, and ameliorate lipid peroxidation [38]. FA was shown to modulate nuclear factor erythroid 2-related factor 2 (Nrf2)/HO-1 pathway, which plays a critical role in mitigating oxidative stress and inflammation in various tissues, including lung, liver, and kidney [38]. Further investigation into whether the Nrf2/HO-1 pathway is involved in the protective effects of FAD012 is ongoing in our laboratory.

Our results further indicated that FAD012 enhanced swallowing reflex function by protecting the dopamine-SP neural mechanism (Figure 7, Figure 8 and Figure 9), which is supposed to play a crucial role in initiating the swallowing reflex. The substantia nigra-striatal dopaminergic system is essential for initiating the swallowing reflex and may serve as a therapeutic target for dysphagia resulting from basal ganglia infarction [21]. Clinically, therapeutic drugs for Parkinson’s disease such as levodopa [39], amantadine [40], and some dopamine receptor agonists [41], have been shown to improve dysphagia by activating the dopaminergic system. Additionally, SP released from sensory nerve endings in the laryngopharyngeal region is critical for initiating the swallowing reflex [19]. FAD012 demonstrated significant efficacy in improving dysphagia at 10 mg/kg/day, with its therapeutic effect comparable to established prescription drugs like perindopril [34] and cilostazol [42], both of which are known for maintaining cerebral blood flow and mitigating ischemic damage. The concentration- and dose-dependent cytoprotective and cerebroprotective effects of FAD012 were demonstrated in both in vitro studies using rat brain microvascular endothelial cells and in vivo models of MCAO/reperfusion injury [23]. In the present study, the 3 mg/kg dose did not produce a statistically significant effect, whereas the 10 mg/kg dose showed clear efficacy. This difference may be attributed to concentration-dependent activation of the underlying pharmacological pathways, with 3 mg/kg possibly falling below the threshold required to elicit a therapeutic response. Furthermore, no signs of toxicity were observed at 10 mg/kg, indicating that this dose is within a safe and effective range. However, the pharmacokinetic properties of FAD012, including its absorption, distribution, metabolism, and excretion, have not yet been fully characterized, and further studies are warranted to elucidate these parameters. Notably, we evaluated the safety of long-term FAD012 administration through plasma biochemistry analysis after 10 weeks of daily oral administration (50 mg/kg). No significant changes were observed in plasma protein levels, liver and kidney function markers, or electrolyte levels, confirming its good tolerability and low toxicity [23]. The low toxicity profile of FAD012 suggests that it could be administered long-term as a prophylactic therapeutic agent, offering a practical approach for managing chronic complications, including dysphagia, with minimal side effects. Given its efficacy in maintaining CBF, protecting the dopamine-SP system, and improving swallowing function, FAD012 appears to be a promising candidate for the long-term prevention and treatment of post-stroke dysphagia. Further studies are needed to clarify the mechanisms underlying its vasodilatory and neuroprotective effects during ischemia, as well as to evaluate its clinical applicability.

## 4. Materials and Methods

### 4.1. Experimental Design

**Figure 10:**
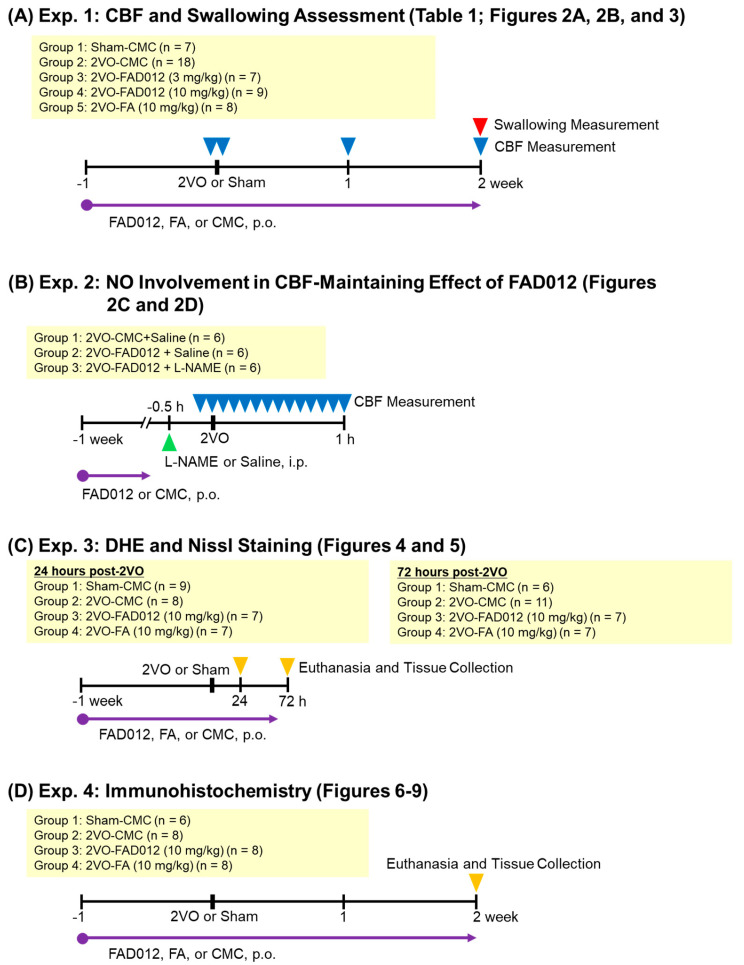
Experimental design of in vivo studies investigating the neuroprotective and swallowing-related effects of FAD012. Four separate experiments (Exp. 1–4) were performed using a rat model of 2VO. (**A**) Exp. 1: Rats were orally administered FAD012, FA, or CMC once daily for 7 days prior to 2VO surgery. CBF was measured immediately before and after 2VO, as well as at 1 and 2 weeks post-2VO. Two weeks after 2VO, swallowing function was evaluated. (**B**) Exp. 2: To investigate the involvement of NO in the FAD012-mediated maintenance of CBF, the NOS inhibitor L-NAME was intraperitoneally administered prior to 2VO, and CBF was monitored continuously before and after 2VO surgery. (**C**) Exp. 3: Histological evaluation was conducted at 24 and 72 h after 2VO. DHE and Nissl staining were performed in the striatum to assess oxidative stress and neuronal cell death, respectively. (**D**) Exp. 4: To explore the mechanism underlying the improvement of swallowing dysfunction, immunohistochemical analysis was performed to detect the expression of cleaved caspase-3, TH, and SP in the striatum, as well as SP expression in the laryngopharyngeal region. Group assignments and the number of animals used in each experiment are indicated in the yellow boxes. Note: A total of 144 rats were used across Exp. 1–4. Due to practical limitations, animals were purchased, assigned, and used separately for each experiment, rather than being randomized and allocated in a single batch. All exclusions occurred after group assignment. A total of 36 rats were excluded prior to analysis due to excessive weight loss, death, or procedural complications (Exp. 1: 10; Exp. 2: 0; Exp. 3: 13; Exp. 4: 13).

As illustrated in Figure 10, four separate in vivo experiments (Exp. 1–4) were conducted to evaluate the neuroprotective and swallowing-related effects of FAD012 in a rat model of 2VO-induced cerebral hypoperfusion. Exp. 1 was designed to assess the effects of chronic oral administration of FAD012 on the reduction of CBF and swallowing dysfunction induced by 2VO (Figure 2A,B and Figure 3). In addition, body weight changes and survival rates were evaluated in this experiment (Table 1). Exp. 2 aimed to investigate the involvement of NO in the FAD012-mediated maintenance of CBF (Figure 2C,D). For this purpose, the NOS inhibitor L-NAME was administered prior to 2VO surgery in selected groups. Exp. 3 and Exp. 4 examined the neuroprotective effects of FAD012 through histological analyses. In Exp. 3, DHE staining (Figure 4) and Nissl staining (Figure 5) were performed in the striatum to evaluate the effects of FAD012 on oxidative stress and neuronal cell death, respectively. These time points (24 and 72 h post-2VO) were selected based on prior studies showing an advanced state of oxidative stress damage and inflammatory response at 24 h, and apoptotic progression at 72 h [43]. Exp. 4 focused on elucidating the mechanism underlying the improvement of swallowing dysfunction. Immunohistochemical analyses were used to assess the expression of cleaved caspase-3 as a marker of apoptosis (Figure 6), TH (Figure 7), and SP (Figure 8) in the striatum as markers of dopaminergic neurotransmission. Additionally, SP expression in the laryngopharyngeal region (Figure 9), which plays a critical role in triggering the swallowing reflex, was evaluated. The group assignments and the number of animals used in each experiment are detailed in Figure 10. In principle, animals were randomly and evenly allocated to each group. Randomization was performed using GraphPad Prism software (version 9.5.0, GraphPad Software, San Diego, CA, USA) based on body weight prior to treatment assignment to minimize selection bias. A larger number of animals were assigned to the Sham and/or 2VO-CMC groups to allow for the optimization of experimental conditions. When the minimum required number of analyzable samples (*n* = 3–5) could not be obtained due to excessive weight loss or death caused by 2VO-induced damage, or due to unforeseen incidents during the experimental procedure, additional animals were included as needed to ensure sufficient statistical power (Figure 10). Outcome assessments, including CBF measurement, swallowing reflex assessment, and histological evaluations, were conducted by investigators blinded to the group allocations to reduce subjective bias.

### 4.2. Animals

A total of 159 male adult Sprague–Dawley rats (10 weeks old) weighing 330–350 g were purchased from Japan SLC, Inc. (Hamamatsu, Japan) and housed under a temperature- (23 ± 0.5 °C) and humidity- (55% ± 10%) controlled environment with a 12/12 h light–dark cycle. The rats were provided with standard rodent chow (CE-2, CLEA Japan, Inc., Tokyo, Japan) and water ad libitum. After a one-week acclimation period, rats were randomly assigned to experimental groups. In a representative experiment (Exp. 1), animals were divided into five groups: sham-CMC (oral administration of saline containing 0.5% CMC (carboxymethyl cellulose, Fujifilm Wako Chemical Co., Osaka, Japan) as a vehicle, followed by sham surgery), 2VO-CMC, FAD012 (3 mg/kg), FAD012 (10 mg/kg), and FA (10 mg/kg; trans-4-hydroxy-3-methoxycinnamic acid, Sigma-Aldrich, St. Louis, MO, USA). All groups underwent the 2VO procedure except the sham group. Group compositions varied slightly across Exp. 1–4 depending on experimental objectives. Detailed group assignments for each experiment are provided in Figure 10. In Exp. 3 and Exp. 4, which involved histological analyses, only the 2VO-FAD012 (10 mg/kg) and 2VO-FA (10 mg/kg) groups were included for comparison, as the higher dose of FAD012 had shown beneficial effects on swallowing dysfunction.

FAD012 was synthesized in our laboratory and fully characterized by ^1^H and ^13^C NMR and MS. The purity of FAD012, as determined by HPLC analysis, was greater than 95% [23,26]. All treatments were administered via gastric intubation at a volume of 0.3 mL/100 g body weight once daily for three weeks, starting one week prior to the 2VO procedure and continuing for two weeks subsequently.

### 4.3. 2VO Procedure [13]

Cerebral hypoperfusion was induced by 2VO as previously described [13]. Rats were anesthetized with isoflurane (5% for induction, 2% for maintenance) and placed in the supine position. A midline neck incision was made to expose the bilateral common carotid arteries. The arteries were carefully separated from the carotid sheath and vagal nerves, then double-ligated and transected between the ligatures. Sham-operated rats underwent the same procedure, except for carotid artery ligation. During the 2VO procedure, the rectal temperature of the rat (36–38 °C) was maintained using a Heating Pad System for Rodents (FHC-HPS, Muromachi Kikai Co., Ltd., Tokyo, Japan). To maintain body fluid volume, saline (1.0 mL/h) was administered intraperitoneally to the rat using a microsyringe infusion pump (KDS100: kdScientific Inc. Holliston, MA, USA).

### 4.4. Measurement of CBF [13]

CBF was measured before and after 2VO treatment. Under isoflurane anesthesia, the rat’s head was fixed in a stereotactic frame (SR-5R-HT, Narishige, Tokyo, Japan) in the prone position, and a midline scalp incision was made to expose the skull. Surficial blood flow was recorded at eight cortical points (+3.0, +1.0, −3.0, and −5.0 mm rostrocaudal from the bregma, and 1.5 mm left and right from the midline) using laser Doppler flowmetry (ATBF-LC1, Unique Medical Co., Ltd., Tokyo, Japan). CBF measurements were taken 30 min prior to 2VO, and 30 min, 7 days, and 14 days post-2VO. CBF values were averaged from both hemispheres. The involvement of NO in the action of FAD012 to maintain CBF during ischemia was investigated using a specific NO synthase inhibitor, L-NAME (N^G^-nitro-L-arginine methyl ester, Sigma-Aldrich, St. Louis, MO, USA). L-NAME (30 mg/kg) was administered intraperitoneally 30 min before 2VO, and time-dependent changes in CBF in the frontal regions (1.0 mm anterior, 1.5 mm lateral to bregma) were measured.

### 4.5. Measurement of Swallowing [13,14]

Swallowing function was assessed following established protocols [13,14]. Fourteen days after 2VO, rats were anesthetized with urethane (1.0 g/kg, i.p.) (ethyl carbamate, Fujifilm Wako Chemical Co., Osaka, Japan) and positioned supine in a stereotactic frame (SR-5R-HT, Narishige, Tokyo, Japan). A midline neck incision was made, and the salivary glands were cauterized at the duct origins to reduce salivation, which could affect swallowing responses. The trachea was intubated to maintain proper ventilation, and a cannula was inserted into the esophagus to drain the stimulating solutions after swallowing. A polyethylene guide tube was secured in the rat’s mouth, allowing a second tube to deliver the stimulating solution directly to the pharynx. A small volume (50 µL) of either distilled water or citric acid solution (1, 3, or 10 mM) was infused into the laryngeal region using an infusion pump (KDS100: kdScientific Inc. Holliston, MA, USA) at a flow rate of 3.3 µL/s for 15 s. The swallowing reflex was monitored by recording EMG activity via a bipolar stainless steel wire electrode placed in the unilateral suprahyoid muscle, along with visual observation of laryngeal movement. MG signals from the mylohyoid muscle were amplified (MEG-5100, Nihon Kohden, Tokyo, Japan) and stored for analysis with a data acquisition system (PowerLab/4s, AD Instruments, Castle Hill, Australia). The latency of the first swallow and the total number of swallows within a 30 s period following infusion were recorded for each rat.

### 4.6. Perfusion Fixation and Coronal Section Preparation [13]

Rats were anesthetized with urethane (1.0 g/kg, i.p.) and transcardially perfused with cold saline to remove blood, followed by perfusion with 0.1 M phosphate buffer (PB) containing 4% paraformaldehyde (PFA) for tissue fixation. Brains were then harvested and post-fixed in 4% PFA at 4 °C overnight. For cryo-embedding, fixed tissues were sequentially immersed in PB containing 10%, 20%, and 30% sucrose. Coronal sections, 20 or 30 μm thick, were prepared using a cryostat (Leica CM3050 S, Bensheim, Germany).

### 4.7. Evaluation of O_2_^−^ Production by Dihydroethidium (DHE) Staining [13]

Intracellular O_2_^−^ generation in the striatum was assessed 24 and 72 h after 2VO using DHE staining. Coronal brain sections (30 µm thick) were incubated with 10 µmol/L DHE (Sigma-Aldrich, St. Louis, MO, USA) in 10 mM phosphate-buffered saline (PBS, pH 7.4) at 37 °C for 30 min. After washing, sections were mounted on slides, and three microscopic fields from the relevant regions were imaged. The fluorescence intensity of oxidized DHE in each field quantified using an All-in-One fluorescence microscope (BZ-X700, Keyence, Osaka, Japan). Histopathological evaluation was performed in a blind manner, without knowledge of the treatment groups.

### 4.8. Nissl Staining [44]

Coronal brain sections (30 µm thick) were washed in 0.1 M PB and incubated in 0.05% toluidine blue solution for 1 min. Excess staining solution was washed off with PB, and the sections were dehydrated sequentially in 70%, 80%, 90%, and 100% ethanol for 1 min each. After clearing with xylene, the sections were mounted on glass slides for microscopic observation. Darkly stained cells, indicative of nuclear aggregation caused by cell death, were counted to evaluate brain tissue damage in the striatum.

### 4.9. Immunohistochemistry [13]

Expression levels of cleaved caspase-3 and TH in the striatum, as well as SP in the striatum and laryngopharyngeal region, were assessed by immunohistochemical staining 14 days post-2VO. Following perfusion with cold saline and fixation with PBS (pH 7.4) containing 4% PFA, coronal brain sections (30 µm thick for the striatum) and sagittal sections (20 µm thick for the laryngopharyngeal region) were incubated in goat serum-blocking solution (S-1000, Vector Laboratories, Youngstown, OH, USA) with 0.3% Triton X-100 in PBS (PBST) for 1 h at room temperature. Sections were then incubated overnight with one of the following primary antibodies: anti-cleaved caspase-3 rabbit monoclonal antibody (1:100; #9664, Cell Signaling Technology, Danvers, MA, USA), anti-TH antibody (1:1000; AB152, Merck Millipore, Darmstadt, Germany), or anti-SP antibody (1:6000; 20064, ImmunoStar, Hudson, WI, USA). After rinsing in PBST, sections were incubated with goat anti-rabbit IgG (H+L) conjugated to Cy3 (1:100; Life Technologies, Rockville, MD, USA) for 1 h at room temperature. Immunofluorescence was visualized and quantified using a fluorescence microscope and associated imaging software. For TH and SP in the striatum, areas with above-threshold fluorescence intensity, detected by a laser at constant intensity, were considered immunopositive and evaluated accordingly.

### 4.10. Statistical Analysis

Statistical differences among groups were assessed using one-way analysis of variance (ANOVA), followed by Tukey’s post hoc multiple-comparison test.

## 5. Conclusions

In conclusion, chronic administration with FAD012 maintained CBF over the 2-week experimental period and prevented the impairment of swallowing reflex in 2VO rats. FAD012 suppressed oxidative cell death and preserved TH expression in the striatum, which correlated with SP expression levels in both the striatum and laryngopharyngeal mucosa. These findings suggest that FAD012 ameliorates swallowing reflex impairment by protecting the nigrostriatal dopamine-SP system from cerebral hypoperfusion-induced damage. Chronic supplementation with FAD012 may offer a promising alternative for the prevention and treatment of cerebral ischemia-induced swallowing dysfunction.

## Figures and Tables

**Table 1:** Weight gain and survival rates of each group at the point of 14th day of ligation of bilateral common carotid arteries (2VO).

Group	Relative Body Weights (%)	Survival Rates (%)
Sham-CMC	112.6 ± 3.2	100 (7/7)
2VO-CMC	99.2 ± 2.2 *	66.7 (12/18)
2VO-FAD012 (3 mg/kg)	102.3 ± 1.3 *	85.7 (6/7)
2VO-FAD012 (10 mg/kg)	98.4 ± 1.9 *	77.8 (7/9)
2VO-FA (10 mg/kg)	98.8 ± 1.6 *	87.5 (7/8)

Values for body weights in each group at 14 days post-2VO are represented as means ± S.E.M. relative to those before 2VO or sham-operation. * *p* < 0.05 vs. Sham-CMC group.

## Data Availability

The original contributions presented in this study are included in the article. Further inquiries can be directed to the corresponding author.

## Abbreviations

The following abbreviations are used in this manuscript:

ANOVA	One-way analysis of variance
AUC	Area under the curve
BBB	Blood–brain barrier
CBF	Cerebral blood flow
CMC	Carboxymethyl cellulose
CPG	Swallowing central pattern generator
DHE	Dihydroethidium
EMG	Electromyogram
FA	Ferulic acid; 4-hydroxy-3-methoxycinnamic acid
FAD012	3,5-Dimethyl-4-hydroxy cinnamic acid
HO-1	Heme oxygenase-1
MCAO	Middle cerebral artery occlusion
L-NAME	N^G^-nitro-L-arginine methyl ester
NO	Nitric oxide
NOS	NO synthase
Nrf2	Nuclear factor erythroid 2-related factor 2
t-PA	Tissue plasminogen activator
PBS	Phosphate-buffered saline
PFA	Paraformaldehyde
ROS	Reactive oxygen species
SP	Substance P
TH	Tyrosine hydroxylase
2VO	Ligation of bilateral common carotid arteries
